# Terrorist Attacks Escalate in Frequency and Fatalities Preceding Highly Lethal Attacks

**DOI:** 10.1371/journal.pone.0093732

**Published:** 2014-04-22

**Authors:** Andy Martens, Raazesh Sainudiin, Chris G. Sibley, Jeff Schimel, David Webber

**Affiliations:** 1 University of Canterbury, Christchurch, New Zealand; 2 University of Auckland, Auckland, New Zealand; 3 University of Alberta, Edmonton, Alberta, Canada; Universidad Carlos III de Madrid, Spain

## Abstract

Highly lethal terrorist attacks, which we define as those killing 21 or more people, account for 50% of the total number of people killed in all terrorist attacks combined, yet comprise only 3.5% of terrorist attacks. Given the disproportionate influence of these incidents, uncovering systematic patterns in attacks that precede and anticipate these highly lethal attacks may be of value for understanding attacks that exact a heavy toll on life. Here we examined whether the activity of terrorist groups escalates–both in the number of people killed per attack and in the frequency of attacks–leading up to highly lethal attacks. Analyses of terrorist attacks drawn from a state-of-the-art international terrorism database (The Global Terrorism Database) showed evidence for both types of escalation leading up to highly lethal attacks, though complexities to the patterns emerged as well. These patterns of escalation do not emerge among terrorist groups that never commit a highly lethal attack.

## Introduction

Using a large state-of-the-art international terrorism database, this research examined whether terrorist incidents that kill a particularly large number of people are preceded by escalation in either the number of people killed per attack or the frequency of attacks. Attacks that result in a large loss of life are especially important to understand. The vast majority of attacks kill one person or no one at all. Yet a small number of more deadly attacks kill many more times the mean number of fatalities per attack (a distribution that can be described with a power law [1]). Further, examining the distribution of fatalities per attack in a comprehensive international terrorism database–the Global Terrorism Database [2]–the number of fatalities in the most deadly 1% of attacks (69,156 fatalities) accounts for 32% of the total number of fatalities recorded in the database (219,078). Thus, a small number of particularly deadly attacks accounts for a disproportionate number of the total fatalities resulting from terrorist attacks.

Considering this distribution, an efficient approach to researching the cost to human life that terrorism exacts may be to treat big attacks–“big” with respect to the number of resulting fatalities–as qualitatively different and to gather information about when these particular attacks tend to occur. Different criteria for the size or importance of a terrorist attack may be considered (e.g., media coverage, financial costs). But certainly a good argument can be made–especially from the perspective of those killed by an attack–that an attack’s most important characteristic is the number of resulting deaths. Here we take this perspective and use the term “big attack” to describe an attack that kills large numbers of people. Recent research [3]–[4] has begun to examine the timing of big attacks by assessing whether, over the course of a terrorist group’s lifespan, the fatalities that result from attacks tend to increase or decrease with each subsequent attack. This work suggests that the number of fatalities per attack does not systematically vary with the number of previous attacks by a group. In other words, the likelihood of a big attack cannot be predicted simply by the number of previous attacks committed by a group.

Given this extant research [3]–[4], which implies big attacks are somewhat randomly scattered across a group’s lifespan, we developed a different analytic strategy for examining their timing. Though big attacks may occur in a relatively random fashion, it may be that small-scale attacks that precede these big attacks occur more systematically. To examine this, we did not examine big attacks themselves, but tested for patterns of escalation in the subset of small-scale attacks that a group commits prior to and leading up to big attacks. In other words, we conducted a series of analyses to describe the attack-trajectories that precede big attacks. Perhaps signals indicative of an escalating trajectory emerge in this series of smaller attacks that precede big attacks.

There are a number of reasons to suspect escalation prior to a big attack. Various lines of psychological research suggest that violence, and perhaps killing in particular, can escalate under certain conditions [5]–[9]. Killing may trigger justification processes, desensitize people, or produce material and/or psychological rewards that increase the likelihood of further killing. It may also be that with experience, group members become more adept at carrying out severe and deadly attacks. A growing ability to carry out deadly attacks may increase the likelihood that the subsequent attack will be even more lethal.

If escalation occurs, it may occur with the number of fatalities per attack and/or may emerge with the frequency of attacks. Further, escalation may occur in a linear or curvilinear fashion (i.e., the slope of escalation may increase as groups approach a big attack). Given these basic possibilities, in progressions of small-scale attacks that lead up to big attacks we tested for the presence of linear and curvilinear escalation in the number of fatalities per attack and the frequency of attacks in a series of Multilevel Random Coefficient Models and Beta Distribution Models.

## Materials and Methods

### Database

We tested for these escalation patterns using the Global Terrorism Database (GTD [2]) produced by the National Consortium for the Study of Terrorism and Responses to Terrorism (accessed on June 13, 2013). The GTD includes broad categories of data for terrorist incidents from 1970 through 2011 (but excluding 1993). The GTD has been developed by merging large existing databases with additional data collection and corroboration directed by the National Consortium for the Study of Terrorism. The data have been gathered from credible U.S. and international online and print news sources. The criteria used for whether to include an event in the database are relatively inclusive. It must be an “intentional act of violence or threat of violence by a non-state actor (i.e., sub-national or non-national).” In addition, it must meet two of the following three criteria: (1) The act was aimed at attaining a political, economic, religious, or social goal; (2) the act included evidence of an intention to coerce, intimidate, or convey a message to a larger audience (or audiences) other than the immediate victims; (3) the act was outside the context of legitimate warfare activities (e.g., the act deliberately targeted civilians or non-combatants). Further information about the GTD’s development and contents can be found at http://www.start.umd.edu/gtd/downloads/Codebook.pdf.

### Data Preparation: Linear and Quadratic Modeling

For our analyses we required that the group responsible for the attack be known. Thus, we omitted attacks for which the group name was listed as “unknown” in the database. In addition, we omitted incidents that the database coded with obvious catch-all labels that do not represent actual organizations (e.g., group labels of “Other,” “Extremists,” “Terrorists,” and “Guerillas”). For analysis, we also required that the date be known (year, month, and day) and that the number of victim fatalities be known. The database does not report how many victims were killed in each attack but it does report the total number of fatalities per attack, and the number of terrorist members killed in the attack. Thus we computed the number of victim fatalities by subtracting the number of terrorist members killed from the total number of fatalities in each attack.

We examined escalation in the progression of small-scale attacks leading up to big attacks. These within-group progressions frequently consist of multiple attacks on the same day. However, the database does not provide information about the chronology of attacks within days. Given the absence of information about within-day timing of attacks, we merged attacks that each group committed on the same day. For example, if a group committed four attacks on one day that killed 1, 0, 3, and 7 people, we re-coded these four events as a single event that killed 11 people. In sum, we recoded and now refer to multiple attacks committed by the same group on one day as one attack. A drawback of this approach is that our measure of fatalities per attack can be influenced by the frequency of attacks on the same day. Keeping this in mind–that the distinction between frequency of attacks and fatalities per attack is not necessarily clear cut–we nevertheless felt that without within-day temporal resolution, merging same-day attacks was the most sensible way to deal with these data. Merging same-day attacks (for which the database can provide the date, group, and victims killed) left 37,092 attacks for analyses.

**Figure 1:**
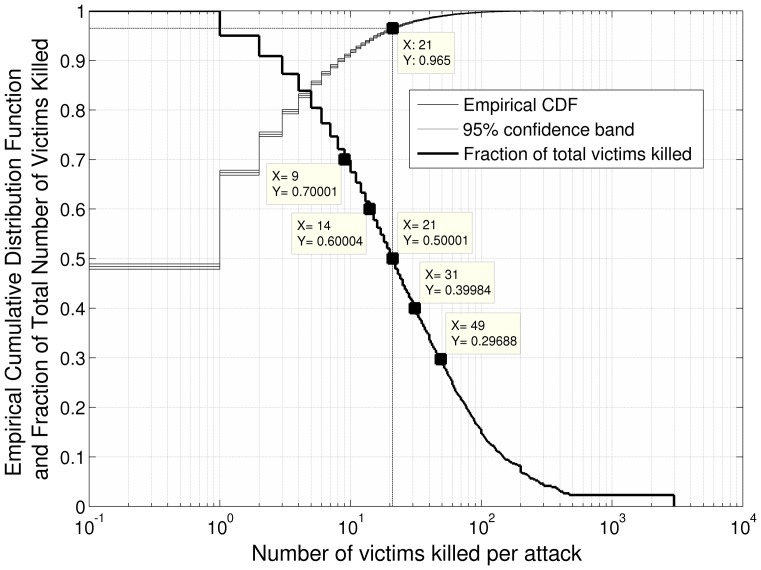
Thresholds used to define big attacks. The bolded line shows the percentage of total fatalities accounted for at different thresholds. For example, attacks killing 49 or more people account for 30% of fatalities from all terrorist attacks combined. Attacks killing 21 or more people account for 50% of all fatalities. We also plot the empirical cumulative distribution function. This shows, for example, that nearly 50% of all terrorist attacks results in zero fatalities, and approximately 67% result in 0 or 1 fatalities. Only 3.5% of attacks results in 21 or more fatalities (i.e., 96.5% of attacks result in fewer than 21 fatalities).

Next, to examine escalation along the progression of small-scale attacks leading up to big attacks, we operationally defined a “big attack.” To do so, we rank ordered the 37,092 attacks in our new dataset by their number of fatalities and then plotted this sequence of attacks against the cumulative percentage of all fatalities in the dataset. In other words, the most deadly attack (2,977 victim fatalities) accounts for 2.0% of the total fatalities from attacks combined in our refined dataset (148,569), the top two most deadly attacks (4,157) account for 2.8% of all fatalities combined, and so on. Figure 1 shows this relationship (along with a standard empirical cumulative distribution function).

We defined a “big attack” by determining the threshold above which attacks cumulatively account for 50% of all fatalities. As can be seen in Figure 1, attacks that kill 21 or more people account for 50% of the total number of fatalities. These attacks comprise just 3.5% of the 37,092 attacks. Thus, this fits our conceptualization of big attacks: a small group of attacks that accounts for a disproportionate number of deaths. The average number of fatalities resulting from a big attack is 52.40 people (*SD* = 94.80, *range* = 21–2,977). We later examine alternative thresholds as well.

**Figure 2:**
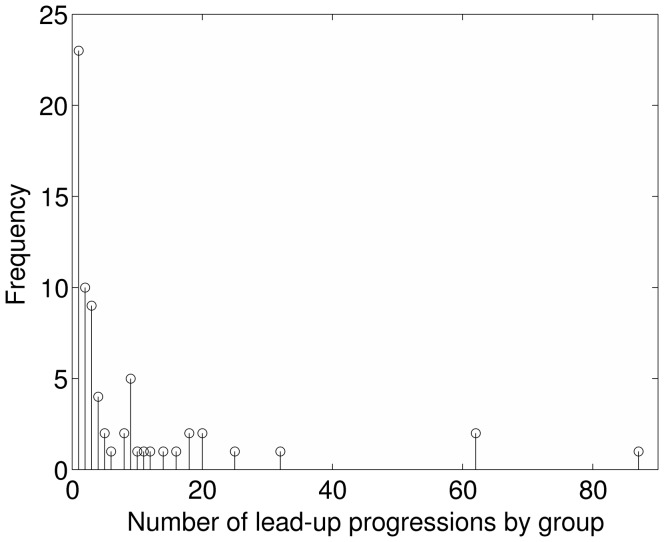
Number of lead-up progressions by group. This distribution of the number of lead-up progressions per group. For example, twenty-two groups committed just one lead-up progression and eleven groups committed just two lead-up progressions.

After defining a big attack, we selected only incidents committed by groups that are included in an official group list on the GTD website (http://www.start.umd.edu/gtd/search/BrowseBy.aspx?category=perpetrator, accessed June 13, 2013). Then, within each terrorist group we found all sets of three or more small-scale incidents (killing fewer than 21 people) that occurred in succession in between big attacks. We term each of these sets of consecutive small-scale incidents a “lead-up progression.” (Given that the GTD had no information for 1993, we did not allow lead-up progressions to span 1993.) As an example, imagine that a hypothetical terrorist group commits 17 attacks: first they commit five small attacks, then a big attack, then ten small attacks, then a big attack, then twelve small attacks, and then a big attack. This group would include two lead-up progressions, the first consisting of ten attacks and the second consisting of twelve attacks. Of the 2,849 groups in our dataset, 68 groups contained at least one lead-up progression. The remainder contained no lead-up progressions either because the group did not commit a big attack or because a series of three or more consecutive small attacks did not fall between big attacks committed by the group. These 68 groups were as follows: Abu Nidal Organization (ANO), Abu Sayyaf Group (ASG), African National Congress (South Africa), Allied Democratic Forces (ADF), Al-Qa’ida, Al-Qa’ida in Iraq, Al-Qa’ida in the Arabian Peninsula (AQAP), Al-Qa’ida in the Lands of the Islamic Maghreb (AQLIM), Al-Shabaab, Ansar al-Sunna, Armed Islamic Group (GIA), Boko Haram, Caucasus Emirate, Communist Party of India - Maoist (CPI-M), Communist Party of Nepal- Maoist (CPN-M), Democratic Revolutionary Alliance (ARDE), Farabundo Marti National Liberation Front (FMLN), Front for the Liberation of Lebanon from Foreigners, Guerrilla Army of the Poor (EGP), Hamas (Islamic Resistance Movement), Haqqani Network, Hizballah, Inkatha, Freedom Party (IFP), Irish Republican Army (IRA), Islamic State of Iraq (ISI), Jemaah Islamiya (JI), Jundallah, Khalistan Liberation Force, Khmer Rouge, Kurdistan Workers’ Party (PKK), Lashkar-e-Jhangvi, Lashkar-e-Taiba (LeT), Liberation Tigers of Tamil Eelam (LTTE), Lord’s Resistance Army (LRA), M-19 (Movement of April 19), Maoist Communist Center (MCC), Misurasata Indian Organization, Moro Islamic Liberation Front (MILF), Moro National Liberation Front (MNLF), Mozambique National Resistance Movement (MNR), Muslim Brotherhood, Muttahida Qami Movement (MQM), National Democratic Front of Bodoland (NDFB), National Liberation Army of Colombia (ELN), National Socialist Council of Nagaland, National Union for the Total Independence of Angola (UNITA), New People’s Army (NPA), Nicaraguan Democratic Force (FDN), Nicaraguan Resistance, Palestine Liberation Organization (PLO), People’s Liberation Front (JVP), People’s War Group (PWG), Rahanwein Resistance Army (RRA), Revolutionary Armed Forces of Colombia (FARC), Revolutionary United Front (RUF), Riyadus-Salikhin Reconnaissance and Sabotage Battalion of Chechen Martyrs, Salafist Group for Preaching and Fighting (GSPC), Sandinista National Liberation Front (FSLN), Shining Path (SL), Simon Bolivar Guerrilla Coordinating Board (CGSB), Sudan People’s Liberation Army (SPLA), Taliban, Tawhid and Jihad, Tehrik-i-Taliban Pakistan (TTP), Tupac Amaru Revolutionary Movement (MRTA), Uganda People’s Army, United Liberation Front of Assam (ULFA), United Self Defense Units of Colombia (AUC). Lead-up progressions consisted of, on average, 18.73 attacks (*SD* = 26.16; *range* = 3–324). A histogram of the number of lead-up progressions per group is shown in Figure 2.

Within each lead-up progression, we rank ordered each incident in ascending temporal order, starting with 0 (i.e., 0 = the first incident in the progression, 1 = the second incident, 2 = the third incident, etc.). However, because lead-up progressions varied substantially in the number of incidents they contained, we scaled the rank-ordered incidents within each lead-up so that 0 always represented the first attack in the lead-up and 1 always represented the last attack in the lead-up (i.e., the attack just prior to big attack). This was accomplished by dividing the rank-ordered number by the total number of incidents in that lead-up progression. This scaling allowed us to equate the first and final attacks in all lead-up progression regardless of how many attacks they contained. For example, the scaling equates incident 30 in a 30-incident lead-up progression with incident 5 in a 5-incident lead-up progression (they are both coded as “1”). Our models therefore rescale time of events leading up to large attacks and only preserve their relative ordering by a uniform position of the unit interval. Thus, we were able to examine escalation in fatalities and attack-frequency from groups’ first to final attack in lead-up progressions, no matter the number of attacks in each progression. We accomplished this by examining the relationships between these scaled ranking and fatalities and between the scaled ranking and time between attacks. Further, we examined curvilinear (quadratic) escalation by squaring the scaled rankings before examining the relationships with fatalities and with time between attacks.

## Results

### Escalation: Fatalities per Attack

#### Basic analysis

First we tested for linear and curvilinear escalation in the number of fatalities per attack over the course of lead-up progressions (i.e., a series of three or more consecutive small-scale attacks that fall between big attacks). We constructed a Multilevel Random Coefficient Model (MRCM) with data from terrorist attacks (Level 1, n = 10,285) nested within lead-up progressions (Level 2, n = 549), nested within terrorist groups (Level 3, n = 68). This modeled each set of attacks in the lead-up to a big attack as its own distinct sequence, controlling for attacks made by the same group. We modeled all effects as random, which allowed parameters to vary across Levels 2 and 3 (i.e., across lead-up progressions and terrorist groups). Including these random effects allows for modeling the slopes and intercepts while recognizing that they may differ across different lead-up progressions and groups. These differences are weighted by their reliability when estimating the average effect (for an in-depth explication of MRCM, see Raudenbush and Bryk’s *Hierarchical linear models* [10]).

Our MRCM was specified as follows:
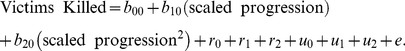
(1)


In Equation 1, the number of victims killed was modeled as a function of an intercept (*b_00_*) which represented the mean number killed during the first incident in a scaled progression (i.e., scaled from 0 to 1), a slope representing the linear scaled progression in victims killed (*b_10_*), and a slope representing the quadratic change in victims killed over the scaled lead-up progression (*b_20_*). The terms *r*_0_, *r*_1_, and *r*_2_ represent the intercept, linear, and quadratic slopes at Level 2. The terms *u_0_*, *u_1_*, and *u_2_* represent the intercept, linear, and quadratic slope at Level 3. Linear and quadratic progressions were uncentered.

**Figure 3:**
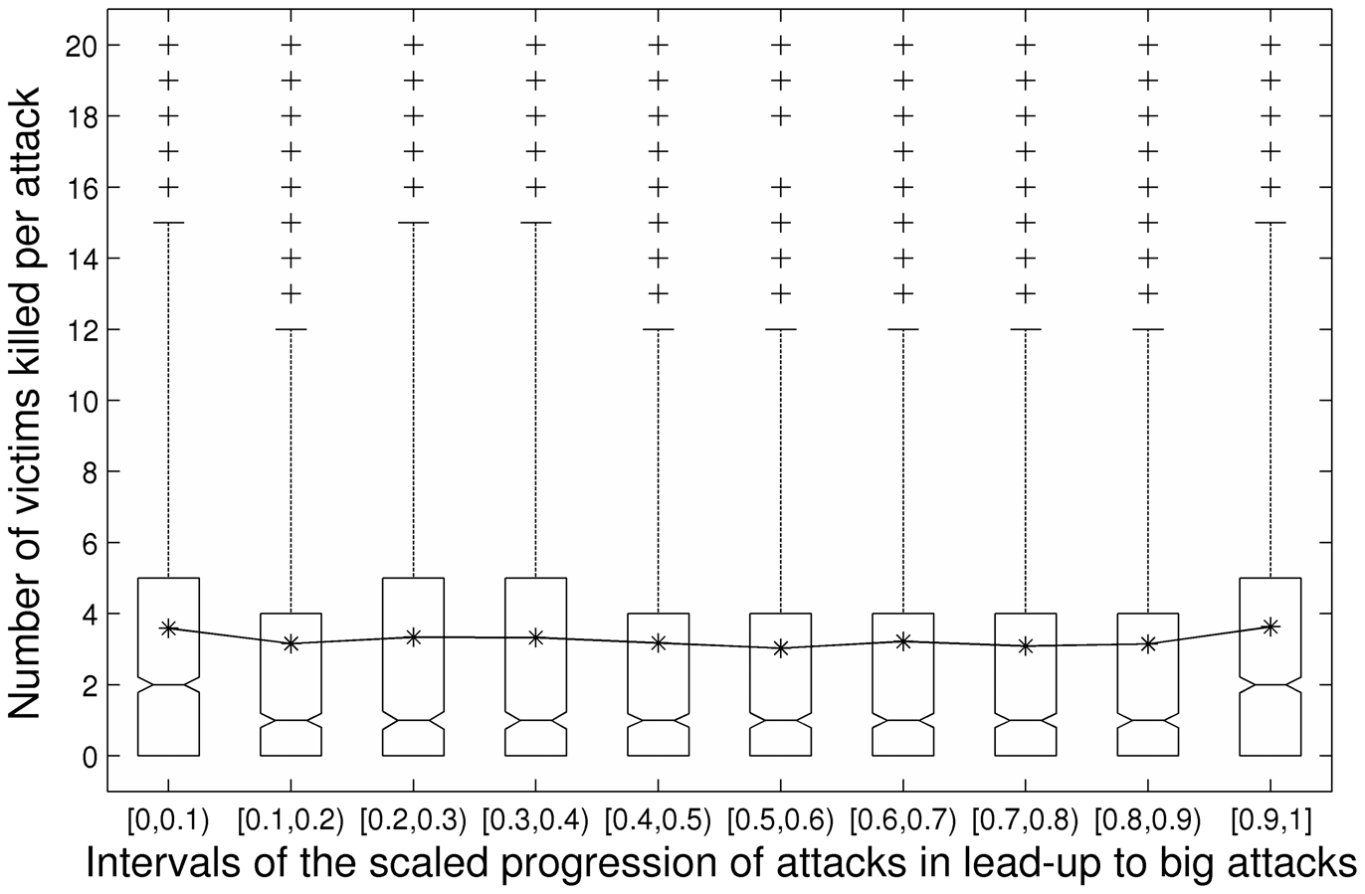
Box plot depicting fatalities per attack over the course of lead-up progressions. Victim fatalities at 10 intervals over the course of the scaled lead-up progressions.

**Figure 4:**
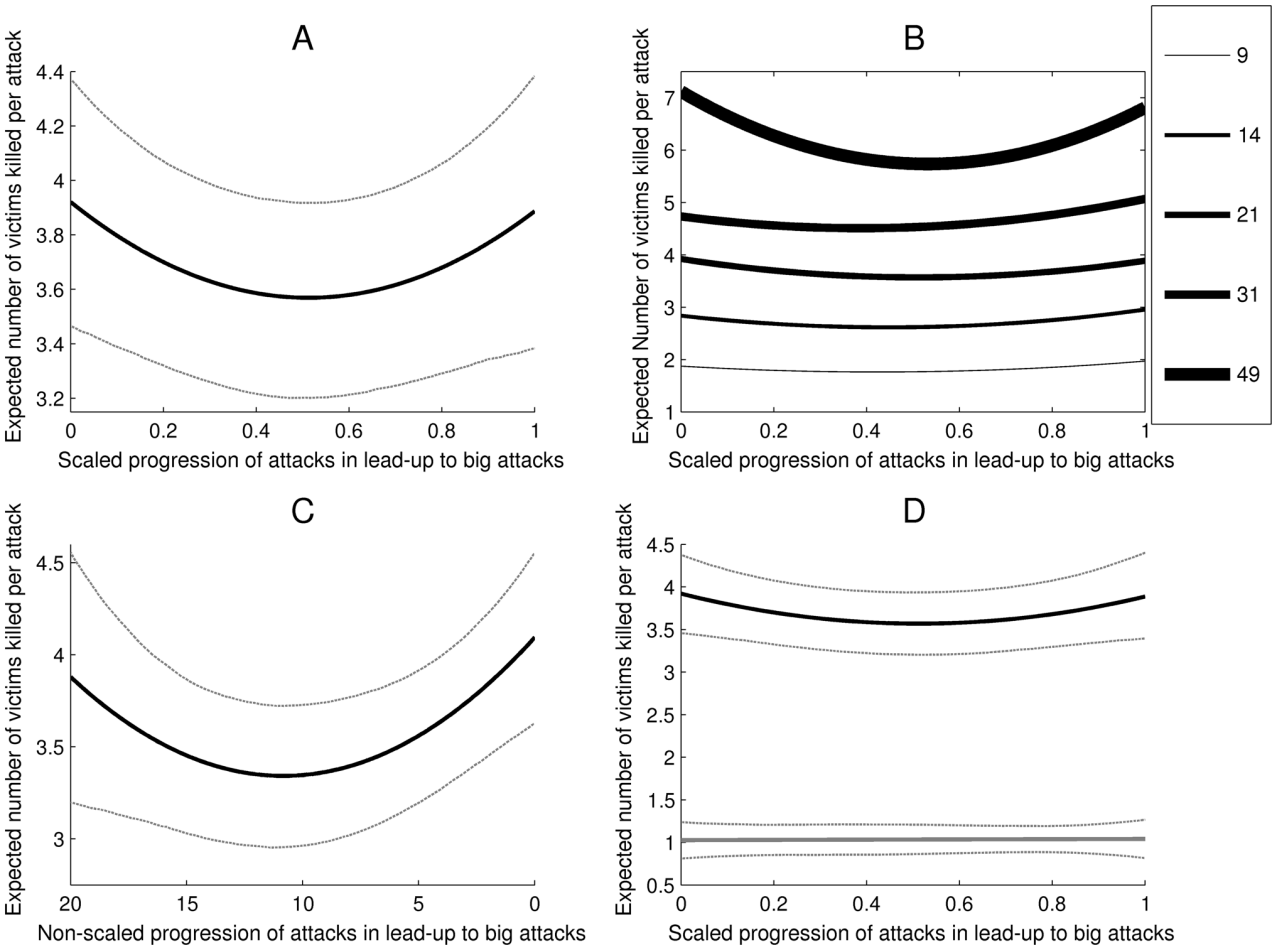
Escalation of victim fatalities per attack. [**A**] Victim fatalities as a function of scaled progression of attacks that lead up to big attacks (i.e., attacks that result in 21 or more victim fatalities). The x-axis value of 0 represents the first incident; 1 represents the incident just prior to a big attack. The dotted lines give the 95% pointwise confidence bands. [**B**] Victim fatalities by scaled lead-up progression using five definitions of a big attack (9, 14, 21, 31, and 49 or more fatalities). [**C**] Victim fatalities by non-scaled lead-up progression. The x-axis value of 19 represents 20 attacks prior to the next big attack; 0 represents the incident just prior to the next big attack. The dotted lines give the 95% pointwise confidence bands. [**D**] Victim fatalities by scaled progression of attacks among groups that never commit a big attack (in grey). The x-axis value of 0 represents a group’s first incident; 1 represents a group’s final incident. For comparison, we also plot (in black) the progression of fatalities leading up to attacks resulting in 21 or more fatalities (from Panel A). The dotted lines give the 95% pointwise confidence bands.



The linear slope over the course of the scaled progression was significant and negative in direction (*b_10_* = −1.37, *SE* = 0.60, *t* = −2.28, *p* = .03; we used *p* = .05 as our threshold for statistical significance in these MRCM analyses). This indicates that, adjusting for the quadratic progression of change, there was a linear decrease in the number of people killed in each sequential attack in the lead-up to big attacks. However, the linear slope should not be interpreted in isolation from the quadratic effect. The quadratic slope indicated significant positively accelerating growth in the numbers killed in sequential attacks preceding big attacks (*b_20_* = 1.34, *SE* = 0.61, *t* = 2.19, *p* = .02). We graphed the raw data in a box plot in Figure 3. Then we graphed the equation comprised of the linear and quadratic functions in Panel A of Figure 4. The dotted lines represent 95% pointwise confidence bands. Our model indicates that the number of victims killed follows a pattern in which there is a slight initial sequential decrease in the number killed. The predicted number of people killed then begins to increase with each small-scale attack prior to a big attack. This function is specified in Equation 2.

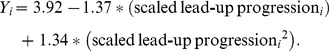
(2)The random effects for the linear and quadratic slopes at Level 2 were both significant, indicating that while these slopes provided the best fitting average trajectory, there was also variation in the progression of people killed in attacks across different lead-up progressions (*χ^2^*(481) = 717.04, *p*<.001; and *χ^2^*(481) = 709.15, *p*<.001, respectively). The random effects for the linear and quadratic slopes at Level 3 were not significant (*χ^2^*(67) = 68.74, *p* = .42; and *χ^2^*(67) = 63.16, *p*>.50, respectively). This suggests that there were no reliable differences in the linear and quadratic rate of progression for different groups when also accounting for variation attributable to differences across lead-up progressions. However, had we been able to access data from a larger number of terrorist groups, we may well have been able to detect more subtle differences in progression rates between groups. Further, these Level 3 effects were obtained while statistically accounting for significant differences between progressions.

We also constructed the MRCM again with a threshold of 21 but excluded outliers–those events with fatalities that exceeded the mean by three or more standard deviations (i.e., greater than 16 fatalities). This model included examined 10,030 terrorist attacks (Level 1) nested within 549 lead-up progressions (Level 2), nested within 68 terrorist groups (Level 3). Essentially mirroring the results when the outliers were included, the linear slope for scaled progression was significant and negative in direction (*b_10_* = −1.22, *SE* = 0.43, *t* = −2.83, *p*<.01) while the quadratic slope indicated significant positively accelerating growth in the numbers killed in sequential attacks preceding a big attack (*b_20_* = 1.27, *SE* = 0.43, *t* = 2.95, *p*<.01).

#### Escalation analysis using alternative definitions of a big attack

The threshold for a big attack was set at 21 fatalities because at this threshold big attacks account for 50% of all fatalities. However, we repeated the above MRCM analysis four more times but adopting different thresholds that account for 30%, 40%, 60%, and 70% of all fatalities. The thresholds that corresponded to these percentages were 9, 14, 31, and 49 fatalities. Panel B of Figure 4 plots the functions for these alternative lead-ups derived using these thresholds. As can be seen, similar patterns emerge in lead-ups to big attacks whether big attacks are defined more conservatively or more liberally.

The MRCM with a threshold of 49 fatalities consisted of 6,333 terrorist attacks (Level 1) nested within 181 lead-up progressions (Level 2), nested within 29 terrorist groups (Level 3). The linear slope for the scaled progression was significant and negative in direction (*b_10_* = −5.20, *SE* = 2.35, *t* = −2.21, *p* = .04) while the quadratic slope indicated significant positively accelerating growth in the numbers killed in sequential attacks in between big attacks (*b_20_* = 4.89, *SE* = 2.04, *t* = 2.39, *p* = .02). The MRCM with a threshold of 31, consisted of 8,661 attacks (Level 1) nested within 346 lead-up progressions (Level 2), nested within 45 terrorist groups (Level 3). The linear slope for the scaled progression was not significant but was negative in direction (*b_10_* = −1.15, *SE* = 1.05, *t* = −1.11, *p* = .28) while the quadratic slope was likewise not significant but was positive in direction (*b_20_* = 1.49, *SE* = 1.05, *t* = 1.41, *p* = .17). The MRCM with a threshold of 14 consisted of 11,712 attacks (Level 1) nested within 860 lead-up progressions (Level 2), nested within 87 terrorist groups (Level 3). The linear slope for the scaled progression was significant and negative in direction (*b_10_* = −0.99, *SE* = 0.31, *t* = −3.25, *p*<.01) while the quadratic slope was significant and positive (*b_20_* = 1.11, *SE* = 0.29, *t* = 3.81, *p*<.01). The MRCM with a threshold of 9 consisted of 13,707 attacks (Level 1) nested within 1,142 lead-up progressions (Level 2), nested within 110 terrorist groups (Level 3). The linear slope for the scaled progression was not significant but was negative in direction (*b_10_* = −0.53, *SE* = 0.29, *t* = −1.84, *p* = .07) while the quadratic slope indicated significant positively accelerating growth in the numbers killed (*b_20_* = 0.63, *SE* = 0.26, *t* = 2.37, *p* = .02).

#### Escalation analysis using non-scaled lead-up progression

Thus far we have examined escalation over the course of scaled lead-up progressions (progressions placed uniformly on a scale from 0 to 1). This allowed us to examine escalation from a group’s first attack to final attack in a lead-up progression, no matter the number of attacks in the progression. The approach we have adopted and the results from these analyses do not indicate the actual number of incidents over which escalation tends to occur. As a consequence, we analyzed the relationship between fatalities and the non-scaled group of incidents leading up to big attacks. To do this we rank-ordered attacks in lead-up progressions in reverse chronological order. We labeled the final attack in a progression (just prior to a big attack) as 0, the attack just prior to this attack as 1, the attack just prior to that attack as 2, etc. Further, we only included attacks from the lead-up progressions if they were within 20 attacks of the big attack.

The MRCM consisted of data from 6,193 terrorist attacks (Level 1) nested within 549 lead-up progressions (Level 2), nested within 68 terrorist groups (Level 3). The linear slope was significant and negative in direction (*b_10_* = −0.14, *SE* = 0.04, *t* = −3.15, *p*<.01). The quadratic slope was significant and positive (*b_20_* = 0.01, *SE* = 0.002, *t* = 2.66, *p* = 0.01). As can be seen in Panel C of Figure 4, this model indicated that fatalities tended to begin escalating only once groups were within approximately 11 incidents of a big attack. The random effects for the linear and quadratic slopes at Level 2 were both significant (*χ^2^*(481) = 601.00, *p*<.001; and *χ^2^*(481) = 597.07, *p*<.001, respectively); the random effects for the linear and quadratic slopes at Level 3 were not significant (*χ^2^*(67) = 71.09, *p* = .34; and *χ^2^*(67) = 77.91, *p* = .17, respectively).

#### Terrorist groups that do not commit a big attack

The above analyses depict the progression of fatalities leading up to big attacks. However, many groups never commit a big attack and thus their entire series of attacks does not lead up to a big attack. We tested for linear and curvilinear escalation in fatalities among these groups to ascertain whether the pattern that emerges above for progressions leading up to a big attack holds among progressions that do not lead up to a big attack. To do so we scaled the entire series of incidents of each group that never committed a big attack from 0 (their first incident) to 1 (their final incident reported in the database). Only groups with three or more attacks were included in the analysis. Then we constructed a two-level random coefficient model with terrorist attacks (Level 1, n = 6,904) nested within terrorist groups (Level 2, n = 291). The model did not hold. The linear and quadratic progressions were both non-significant (*b_10_* = 0.017, *SE* = 0.37, *t* = 0.05, *p* = .960; and *b_20_* = −0.002, *SE* = 0.39, *t* = −0.01, *p* = .995, respectively). Thus we observed no escalation among groups that do not commit a big attack. In Panel D of Figure 4 we graphed this function and for comparison again graphed the function resulting from progressions that lead up to big attacks from Panel A of Figure 4.

### Escalation: Frequency of Attacks

#### Basic analysis

**Figure 5:**
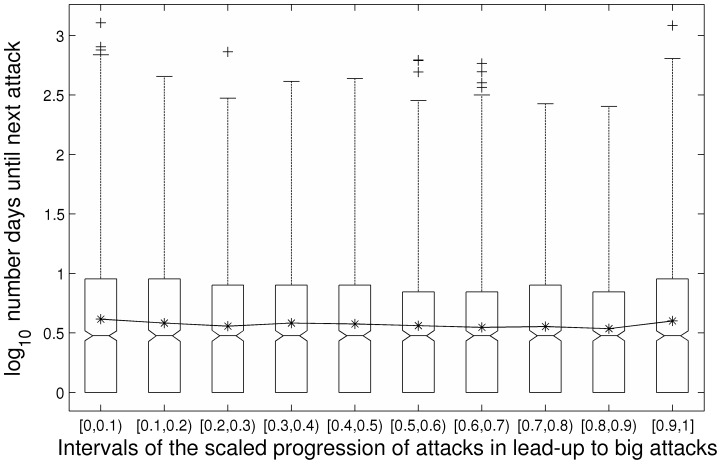
Box plot depicting frequency of attacks over the course of lead-up progressions. Days until next attack at 10 intervals over the course of the scaled lead-up progressions.

**Figure 6:**
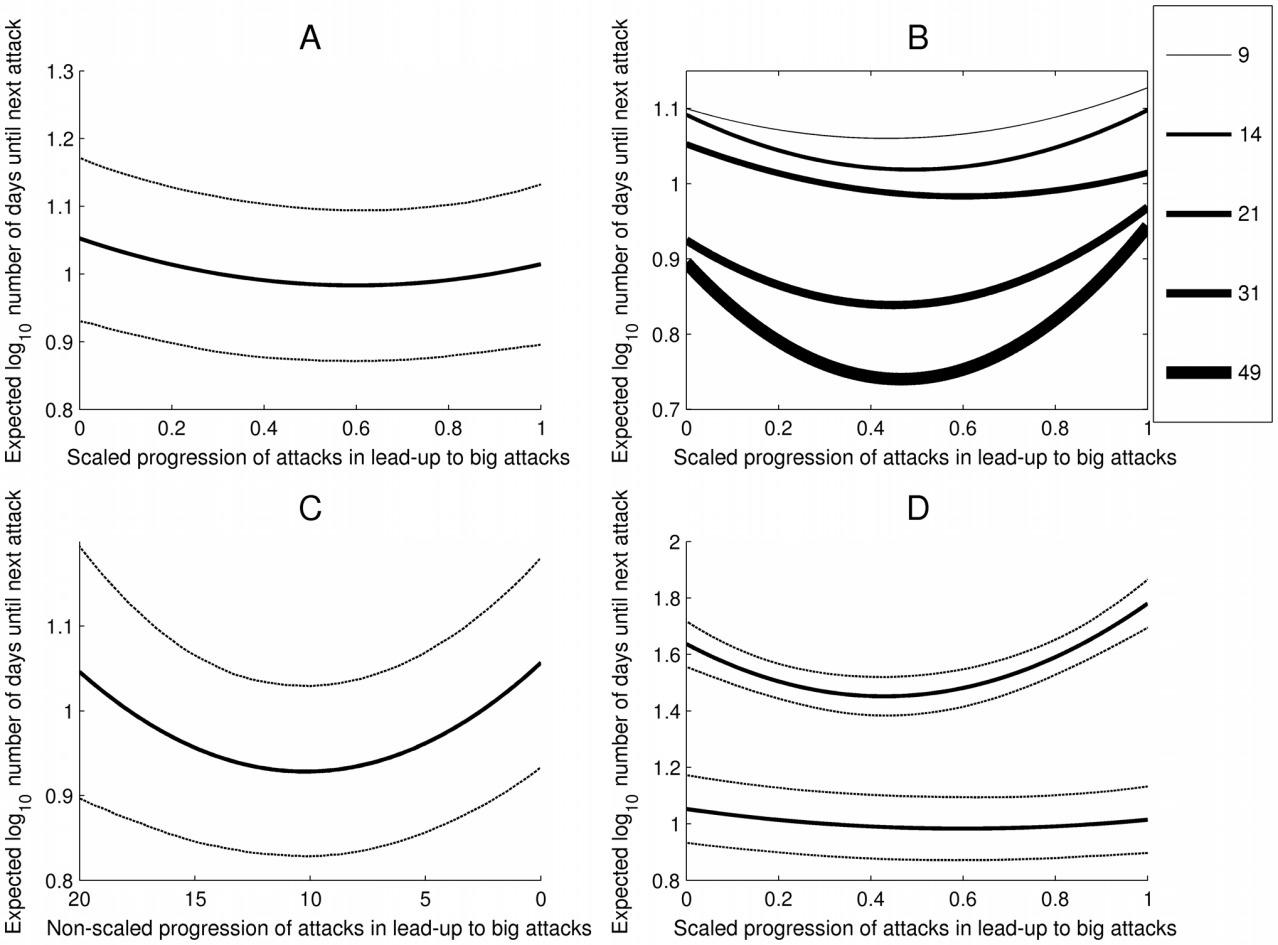
Escalation of frequency of attacks. [**A**] Days until next attack as a function of scaled progression of attacks that lead up to big attacks (that result in 21 or more victim fatalities). The x-axis value of 0 represents the first incident; 1 represents the incident just prior to a big attack. The dotted lines give the 95% pointwise confidence bands. [**B**] Days until next attack by scaled lead-up progression using five definitions of a big attack (9, 14, 21, 31, and 49 or more fatalities). The x-axis value of 0 represents the first incident; 1 represents the incident just prior to a big attack. [**C**] Days until next attack by non-scaled lead-up progression. The x-axis value of 19 represents 20 attacks prior to the next big attack; 0 represents the incident just prior to the next big attack. The dotted lines give the 95% pointwise confidence bands. [**D**] Days until next attack by scaled progression of attacks among groups that never commit a big attack (in grey). The x-axis value of 0 represents a group’s first incident; 1 represents a group’s final incident. For comparison, we also plot (in black) the days until the next attack in lead-up progressions shown in Panel A. The dotted lines give the 95% pointwise confidence bands.



In addition to examining escalation in fatalities over the course of scaled lead-up progressions, we examined linear and curvilinear escalation in the frequency of attacks over the course of lead-up progressions. We constructed a 3-level MRCM just as before except used the log transformed number of days until the next attack as the dependent measure (we used log_10_). The linear slope for scaled lead-up progression was significant and negative in direction (*b_10_* = −0.23, *SE* = 0.08, *t* = −2.77, *p*<.01). This indicates that there was a linear decrease in the log number of days between each sequential attack in the lead-up to a big attack. The quadratic slope indicated positively accelerating growth in the log number of days between attacks in sequential attacks preceding a big attack (*b_20_* = 0.19, *SE* = 0.09, *t* = 2.20, *p* = .03). We graphed the raw data using a box plot in Figure 5 and then graphed the equation comprised of the linear and quadratic functions together in Panel A of Figure 6. As can be seen, there is a sequential decrease in the days until the next attack over the first half of the number of events in the average lead-up progression, but an increase in days until the next attack over the second half of the events in the average lead-up progression. This function is specified in Equation 3:

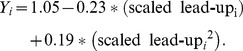
(3)The random effects for the linear and quadratic slopes at Level 2 were both significant (*χ^2^*(481) = 734.66, *p*<.001; and *χ^2^*(481) = 692.40, *p*<.001, respectively). The random effects for the linear and quadratic slopes at Level 3 were significant as well (*χ^2^*(67) = 105.78, *p*<.01; and *χ^2^*(67) = 104.14, *p*<.01, respectively).

#### Escalation using varying definitions of big attack

As with fatalities, we examined escalation in frequency using big-attack thresholds of 49, 31, 14, and 9 fatalities. Panel B of Figure 6 plots the functions for these alternative lead-up progressions derived using thresholds of 49, 31, 21, 14, and 9 fatalities. Somewhat similar patterns emerge in lead-ups to big attacks whether big attacks are defined more conservatively or more liberally. For the MRCM with a threshold of 49 fatalities, the linear slope for the scaled progression was significant and negative in direction (*b_10_* = −0.66, *SE* = 0.21, *t* = −3.10, *p*<.01) while the quadratic was significant in the positive direction (*b_20_* = 0.71, *SE* = 0.25, *t* = 2.87, *p*<.01). With a threshold of 31 fatalities, the linear slope for the scaled progression was significant and negative in direction (*b_10_* = −0.38, *SE* = 0.16, *t* = −2.36, *p* = .02) while the quadratic was significant in the positive direction (*b_20_* = 0.43, *SE* = 0.16, *t* = 2.66, *p* = .01). With a threshold of 14 fatalities, the linear slope for the scaled progression was significant and negative in direction (*b_10_* = −0.29, *SE* = 0.07, *t* = −4.21, *p*<.01) while the quadratic was significant and positive in direction (*b_20_* = 0.30, *SE* = 0.08, *t* = 3.92, *p*<.01). With a threshold of 9 fatalities, the linear slope for the scaled progression was significant and negative in direction (*b_10_* = −0.18, *SE* = 0.06, *t* = −3.18, *p*<.01) while the quadratic was significant and positive in direction (*b_20_* = 0.21, *SE* = 0.06, *t* = 3.42, *p*<.01).

#### Escalation analysis using non-scaled lead-up progressions

As with fatalities, we analyzed the relationship between time until the next attack and the non-scaled lead-up progressions of up to 20 attacks. The linear slope was significant (*b_10_* = −0.025, *SE* = 0.005, *t* = −5.48, *p*<.01) as well as the quadratic slope (*b_20_* = 0.001, *SE* = 0.000, *t* = 4.38, *p*<.01). This pattern, graphed in Panel C of Figure 6, is roughly consistent with the overall results presented up to this point. It suggests that the number of days between attacks increases among the last attacks in a lead-up progression.

#### Terrorist groups that do not commit a big attack

As with number of fatalities, we tested for linear and curvilinear escalation in frequency of attacks among groups that never commit a big attack. We constructed a two-level random coefficient model with 6,904 terrorist attacks (Level 1) nested within 291 terrorist groups (Level 2). The linear progression emerged as significant (*b_10_* = −0.86, *SE* = 0.17, *t* = −4.95, *p*<.001) as did the quadratic progression (*b_20_* = 1.01, *SE* = 0.17, *t* = 5.88, *p*<.001). We plot this function in Panel D of Figure 6 next to the function for escalation in frequency leading up to big attacks for comparison. Attacks that do not lead up to a big attack show initial decrease in the number of days between attacks followed by particularly severe increase in the number of days between attacks, as compared to the number of days between attacks observed among attacks that are leading up to a big attack. When comparing these two types of groups, however, we should keep in mind that the graphed progression of groups that never commit a big attack spans the entire life cycle of these groups. Thus this increase in number of days between attacks toward the end of the progression may represent a disbanding or disintegration of the group and the dynamics of these groups during periods in which they are more fully functioning may look different.

### Data Preparation: Beta Distribution Models

To supplement the above analyses, we conducted a second set of analyses using a similar overall approach but with a different modeling tool: a beta distribution. Using a beta distribution we were able to model when in time attacks tend to occur over the course of a progression, relative to the overall duration of the progression. In contrast, the above MRCM analyses could not consider the time of an attack within the progression beyond where it fell in the rank-ordering of attacks. A beta distribution also allowed us to estimate both a de-escalatory parameter immediately following big attacks as well as an escalatory parameter leading up to big attacks. We examined these parameters among small-scale sequences of attacks that occur between big attacks. In addition, after conducting a global analysis across groups, we examined beta distributions separately for each group involved in the global analysis in order to provide a rough indication of each group’s de-escalatory and escalatory components.

To conduct these analyses, we selected all sequences of three or more small-scale attacks (i.e., attacks with 20 or fewer fatalities) that fell between two big attacks (of 21 or more fatalities). Also, given that we were examining individual group patterns in addition to global/average patterns, we analyzed groups with only a reasonably large number of attacks with which to estimate beta distribution parameters. Specifically, we selected only groups with at least 30 small attacks that fell between big attacks (this might consist of more than one progression). Lead-up progressions consisted of an average of 20.6 attacks (*SD* = 28.2; *range* = 3–303).

As in the above set of MCRM models, we scaled the incidents that make up each progression–we put each progression on the same scale from 0 to 1–in order to pool the data from each progression. We then used a beta distribution to model the density of attacks and fatalities per attack over the course of these progressions. However, we were able to preserve more information about the timing of attacks than in the above MRCM analyses in which we simply rank-ordered the attacks. Instead of rank-ordering, we scaled the timing of attack according to their position in their progressions relative to the overall length of their progression. Specifically, for each attack we divided the number of days since the most recent big attack by the entire length of the progression (the number of days between the two big attacks flanking the progression). Using a beta distribution we were then able to examine de-escalation and escalation in (1) the frequency of attacks by examining the density of attacks at different points over inter-big-attack progressions and in (2) fatalities by examining the density of fatalities at these different points.

### Escalation: Frequency of Attacks and Fatalities per Attack

#### Basic analyses

First we tested for de-escalation in attack-frequency following big attacks and escalation of attack-frequency approaching big attacks. We estimated a Beta Distribution Model with 10,261 attacks, 474 inter-big-attack sequences, and 42 different groups. This analysis modeled all attacks in between big attacks according to the probability density function given by the beta distribution. The probability density function for the beta distribution (up to the normalizing constant) is as follows: x^α-1^(1-x)^β-1^. This analysis produces maximum likelihood estimates of the alpha and beta parameters. An alpha parameter estimate below 1 along with a 99% confidence interval that does not include 1 suggests initial de-escalation following a big attack (and an alpha parameter above 1 suggests initial escalation). A beta parameter estimate below 1 along with a 99% confidence interval that does not include 1 suggests escalation towards the end of a progression just prior to a big attack (a beta parameter above 1 suggests de-escalation approaching a big attack).

The results for data pooled across all groups showed estimates of *alpha* = .949, 99% CI [.916, .983], and *beta* = .910, 99% CI [.878, .943]. The upper boundary of the *alpha* CI is less than 1, which suggests a de-escalation of attack-frequency immediately following a big attack. The upper boundary of the *beta* CI is also less than 1, which suggests an escalation in attack-frequency as groups approach a big attack. In other words, we rejected the null hypothesis that *alpha* ≥1 and *beta* ≥1 at the 1% significance level. This pattern is depicted in Panel A of Figure 6. We also present group-specific estimates of the alpha and beta parameters in Table 1. These 42 groups are ranked by their beta (i.e., escalatory) parameter.

**Table 1:** Beta Distribution Parameters by Individual Group.

		Frequency of Attack		Fatalities per Attack	
Rank	Group	β (+/−90% CI)	α (+/−90% CI)	β (+/−90% CI)	α (+/−90% CI)
1	Al-Qa’ida in the Arabian Peninsula (AQAP)	0.389 (+/−0.220)	1.016 (+/−0.290)	0.418 (+/−0.121)	1.107 (+/−0.190)
2	Moro Islamic Liberation Front (MILF)	0.429 (+/−0.106)	0.380 (+/−0.103)	0.462 (+/−0.064)	0.353 (+/−0.056)
3	Hamas (Islamic Resistance Movement)	0.675 (+/−0.106)	0.694 (+/−0.148)	0.749 (+/−0.049)	0.553 (+/−0.059)
4	Simon Bolivar Guerrilla Coordinating Board (CGSB)	0.696 (+/−0.248)	0.779 (+/−0.225)	0.835 (+/−0.151)	0.663 (+/−0.090)
5	United Liberation Front of Assam (ULFA)	0.720 (+/−0.201)	0.850 (+/−0.189)	0.809 (+/−0.111)	0.961 (+/−0.118)
6	Communist Party of India - Maoist (CPI-M)	0.720 (+/−0.089)	1.155 (+/−0.123)	0.684 (+/−0.048)	1.070 (+/−0.069)
7	Muslim Brotherhood	0.752 (+/−0.338)	0.978 (+/−0.376)	0.833 (+/−0.191)	1.269 (+/−0.262)
8	Democratic Revolutionary Alliance (ARDE)	0.773 (+/−0.385)	0.784 (+/−0.368)	0.977 (+/−0.195)	1.016 (+/−0.239)
9	Revolutionary United Front (RUF)	0.775 (+/−0.469)	0.648 (+/−0.364)	1.005 (+/−0.251)	0.503 (+/−0.119)
10	Al-Qa’ida in Iraq	0.806 (+/−0.212)	0.904 (+/−0.225)	0.899 (+/−0.103)	0.882 (+/−0.106)
11	National Liberation Army of Colombia (ELN)’	0.812 (+/−0.117)	0.843 (+/−0.122)	0.781 (+/−0.072)	0.929 (+/−0.085)
12	National Union for the Total Independence of Angola	0.814 (+/−0.158)	1.018 (+/−0.182)	0.845 (+/−0.068)	0.866 (+/−0.077)
13	Tupac Amaru Revolutionary Movement (MRTA)	0.832 (+/−0.250)	1.114 (+/−0.342)	0.636 (+/−0.140)	0.947 (+/−0.239)
14	Nicaraguan Resistance	0.858 (+/−0.235)	0.838 (+/−0.220)	0.931 (+/−0.122)	0.838 (+/−0.104)
15	Kurdistan Workers’ Party (PKK)	0.887 (+/−0.112)	0.845 (+/−0.097)	0.928 (+/−0.049)	0.863 (+/−0.040)
16	Al-Qa’ida in the Lands of the Islamic Maghreb	0.893 (+/−0.216)	0.812 (+/−0.205)	0.613 (+/−0.091)	0.654 (+/−0.084)
17	Liberation Tigers of Tamil Eelam (LTTE)	0.897 (+/−0.067)	0.998 (+/−0.077)	0.896 (+/−0.032)	0.928 (+/−0.034)
18	Tehrik-i-Taliban Pakistan (TTP)	0.898 (+/−0.245)	0.988 (+/−0.243)	0.890 (+/−0.134)	1.138 (+/−0.148)
19	Boko Haram	0.905 (+/−0.311)	0.958 (+/−0.287)	0.868 (+/−0.189)	0.872 (+/−0.159)
20	New People’s Army (NPA)	0.908 (+/−0.090)	0.991 (+/−0.094)	0.896 (+/−0.043)	0.977 (+/−0.046)
21	Nicaraguan Democratic Force (FDN)	0.917 (+/−0.150)	0.975 (+/−0.154)	0.831 (+/−0.063)	0.993 (+/−0.068)
22	M-19 (Movement of April 19)	0.919 (+/−0.148)	0.858 (+/−0.143)	0.850 (+/−0.070)	0.926 (+/−0.076)
23	Lord’s Resistance Army (LRA)	0.961 (+/−0.191)	0.871 (+/−0.165)	0.952 (+/−0.075)	0.751 (+/−0.060)
24	Irish Republican Army (IRA)	0.982 (+/−0.123)	1.056 (+/−0.120)	1.059 (+/−0.086)	1.074 (+/−0.084)
25	Revolutionary Armed Forces of Colombia (FARC	0.983 (+/−0.084)	0.907 (+/−0.075)	1.018 (+/−0.041)	0.950 (+/−0.037)
26	Shining Path (SL)	0.988 (+/−0.068)	1.020 (+/−0.067)	0.922 (+/−0.029)	1.001 (+/−0.030)
27	People’s Liberation Front (JVP)	1.006 (+/−0.211)	0.890 (+/−0.212)	0.896 (+/−0.080)	0.917 (+/−0.099)
28	Taliban	1.035 (+/−0.071)	1.096 (+/−0.075)	1.051 (+/−0.037)	1.062 (+/−0.037)
29	Al-Shabaab	1.038 (+/−0.164)	1.300 (+/−0.182)	0.973 (+/−0.096)	1.130 (+/−0.098)
30	Islamic State of Iraq (ISI)	1.042 (+/−0.440)	1.138 (+/−0.419)	0.816 (+/−0.213)	1.141 (+/−0.216)
31	Mozambique National Resistance Movement (MNR)	1.066 (+/−0.286)	1.057 (+/−0.249)	0.981 (+/−0.102)	1.041 (+/−0.101)
32	Sandinista National Liberation Front (FSLN)	1.087 (+/−0.418)	1.082 (+/−0.384)	1.513 (+/−0.376)	1.639 (+/−0.342)
33	Farabundo Marti National Liberation Front (FMLN)	1.089 (+/−0.080)	1.071 (+/−0.088)	1.176 (+/−0.045)	1.064 (+/−0.045)
34	Moro National Liberation Front (MNLF)	1.100 (+/−0.319)	1.029 (+/−0.369)	0.861 (+/−0.102)	0.968 (+/−0.207)
35	Hizballah	1.109 (+/−0.142)	0.775 (+/−0.120)	1.198 (+/−0.106)	0.872 (+/−0.089)
36	Khmer Rouge	1.130 (+/−0.405)	0.750 (+/−0.271)	0.974 (+/−0.171)	0.881 (+/−0.157)
37	Armed Islamic Group (GIA)	1.211 (+/−0.170)	0.881 (+/−0.161)	0.917 (+/−0.056)	0.825 (+/−0.077)
38	People’s War Group (PWG)	1.335 (+/−0.547)	1.482 (+/−0.366)	1.062 (+/−0.252)	0.871 (+/−0.122)
39	Lashkar-e-Taiba (LeT)	1.421 (+/−0.432)	2.182 (+/−0.684)	1.727 (+/−0.226)	2.465 (+/−0.321)
40	Abu Sayyaf Group (ASG)	1.586 (+/−0.532)	1.983 (+/−0.532)	1.728 (+/−0.358)	2.610 (+/−0.364)
41	Al-Qa’ida	1.593 (+/−0.611)	1.721 (+/−0.648)	2.141 (+/−0.358)	2.002 (+/−0.416)
42	Palestine Liberation Organization (PLO)	3.722 (+/−0.432)	2.264 (+/−0.558)	4.231 (+/−0.420)	2.481 (+/−0.470)

Beta distribution parameters for inter-big-attack sequences by individual terrorist group. Groups are rank-ordered according to their beta coefficient that represents the escalation in frequency of attacks prior to a big attack (defined as attacks resulting in 21 or more fatalities). The beta for fatalities represents the escalation in fatalities per attack prior to a big attack. The alphas represent de-escalation in frequency and fatalities following a big attack.

Next we utilized the beta distribution model to examine fatalities per attack following big attacks and as groups approach big attacks. We accomplished this by modelling an event for each fatality at approximately the time point that the attack occurred (up to the day). Thus as with the above analysis, for an attack with 0 fatalities we modeled 1 event, but for an attack with 1 fatality we modeled 2 events, for an attack with 2 fatalities we modeled 3 events, and so on. At most, one event can occur at any one point on the unit interval under the continuous Beta Distribution. Due to our coarse time measurement scale we can only observe these events up to units of days (our results are robust to uniformly distributing multiple events on the same day to a 24-hour window and subsequently rescaling to the unit interval).

**Figure 7:**
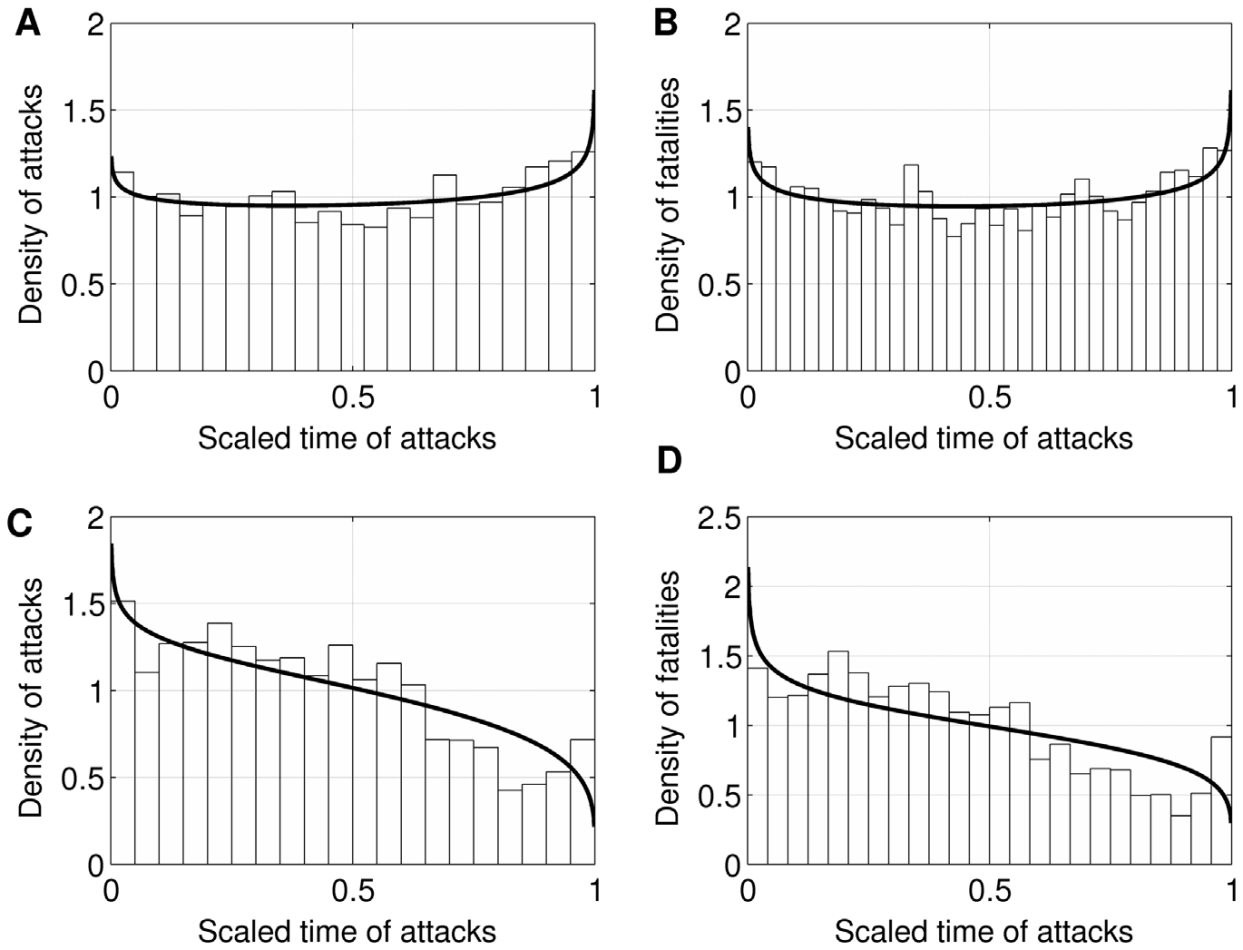
Beta Distribution Models of Escalation. [**A**] Frequency of attacks as a function of inter-big-attack progressions. X-axis values closer to 0 represent attacks towards the beginning of a progression; x-axis values closer to 1 represent attacks towards the end of a progression. Larger y-axis values represent a larger number of attacks at that point during the progression. [**B**] Fatalities per attack as a function of inter-big-attack progressions. X-axis values closer to 0 represent attacks towards the beginning of a progression; x-axis values closer to 1 represent attacks towards the end of a progression. Larger y-axis values represent a larger number of fatalities per attack at that point during the progression. [**C**] Frequency of attacks among groups that never commit a big attack. X-axis values closer to 0 represent attacks towards the beginning of a group’s lifespan; x-axis values closer to 1 represent attacks towards the end of a group’s lifespan. Larger y-axis values represent a larger number of attacks at that point during a group’s lifespan. [**D**] Fatalities per attack among groups that never commit a big attack. X-axis values closer to 0 represent attacks towards the beginning of a group’s lifespan; x-axis values closer to 1 represent attacks towards the end of a group’s lifespan. Larger y-axis values represent a larger number of fatalities per attack at that point during a group’s lifespan.

The results showed *alpha* = .926, 99% CI [.910, .943], and *beta* = .906, 99% CI [.890, .922]. The upper boundaries of the alpha and beta CIs are less than 1 (rejecting the null hypothesis that *alpha* ≥1 and *beta* ≥1 at the 1% significance level). This suggests a de-escalation of fatalities-per-attack immediately following a big attack and escalation of fatalities-per-attack as groups approach a big attack. This pattern is depicted in Figure 7, Panel B. Estimates of alpha and beta parameters for each group are presented in Table 1.

We also examined our statistical power for this fatalities analysis and for the frequency analysis by simulating the 10,261 data points repeatedly 10,000 times from the maximum likelihood estimates of alpha and beta parameters. We then used this simulated data to attempt a rejection of the null hypothesis that alpha and beta are < = 1.0 (the null states that the density distribution is not U-shaped). At the 1% significance level the tests are high in power: 92.66% and 99.96% for the beta distribution analyses of frequency and fatalities, respectively.

#### Terrorist groups that do not commit a big attack

As with our original set of MRCM analyses, we examined the Beta Distribution Model among groups that never commit a big attack and thus whose progressions of attacks do not lead up to a big attack. This model contained 6,160 attacks and 64 different groups. First we examined the frequency of attacks. The results showed *alpha* = .931, 99% CI [.892, .972], and *beta* = 1.240, 99% CI [1.194, 1.289]. The upper boundary of the alpha CI is less than 1, which suggests a de-escalation of attacks immediately following the first attack. The lower boundary of the beta CI is greater than 1, which suggests a further de-escalation of attacks as groups approach their last attack. The escalatory component present in progressions leading up to big attacks is thus absent in these groups who are not leading up to a big attack. This pattern is depicted in Figure 7, Panel C.

Next we examined the number of fatalities per attack with the Beta Distribution Model among the groups that never commit a big attack. The results showed *alpha* = .897, 99% CI [.868, .927], and *beta* = 1.183, 99% CI [1.151, 1.215]. The upper boundary of the alpha CI is less than 1, which suggests a de-escalation of fatalities-per-attack immediately following their first attack. The lower boundary of the beta CI is greater than 1, which suggests a further de-escalation of fatalities-per-attack as groups approach their last attack. Thus the escalatory component present in progressions leading up to big attacks is absent in these groups who are not leading up to a big attack. This pattern is depicted in Figure 7, Panel D.

#### Escalation analysis using alternative definitions of a big attack

**Figure 8:**
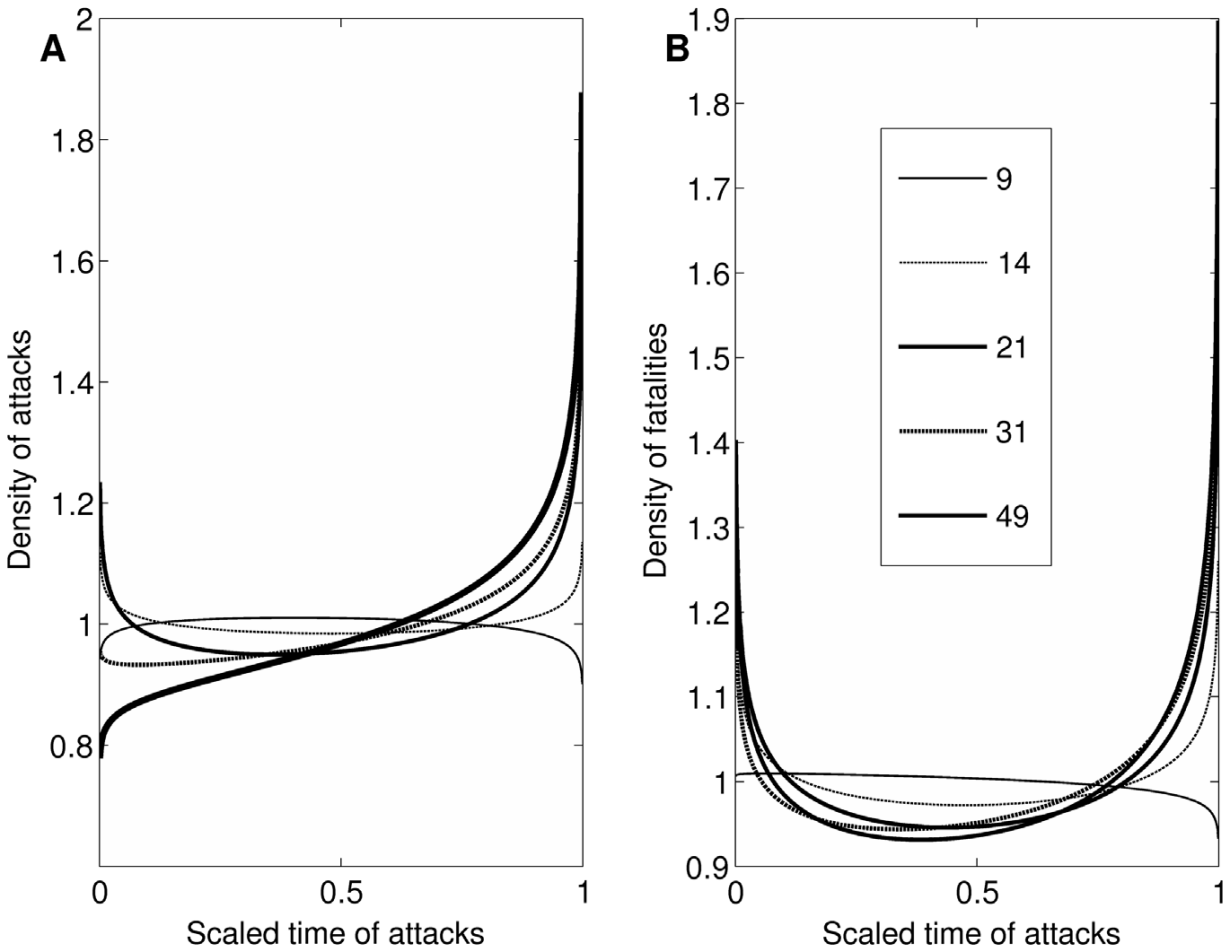
Beta Distribution Models of Escalation at Different Thresholds for Big Attacks. [**A**] Frequency of attacks by inter-big-attack progressions using various definitions of a big attack (9, 14, 21, 31, and 49 fatalities). [**B**] Fatalities per attack by inter-big-attack progressions using various definitions of a big attack (9, 14, 21, 31, and 49 fatalities).

As with the MRCM analyses, we examined the Beta Distribution Model again using the alternative thresholds of 49, 31, 14, and 9. First we examined frequency of attacks using these alternative thresholds. The patterns for each threshold can be seen in Panel A of Figure 8. As the threshold increases, the models show stronger escalatory patterns in frequency of attacks leading up to big attacks (and weaker patterns of de-escalation in frequency of attacks following big attacks). Specifically, using a threshold of 49, *alpha* = 1.023, 99% CI [.981, 1.067], *beta* = .896, 99% CI [.857, .937]; using a threshold of 31, *alpha* = .993, 99% CI [.956, 1.031], *beta* = .916, 99% CI [.881, .952]; using a threshold of 14, *alpha* = .976, 99% CI [.944, 1.010], *beta* = .975, 99% CI [.942, 1.008]; using a threshold of 9, *alpha* = 1.013, 99% CI [.983, 1.045], *beta* = 1.020, 99% CI [.989, 1.052].

We next examined fatalities per attack. As seen in Panel B of Figure 8, as the thresholds increase the models again show stronger patterns of escalation of fatalities-per-attack as groups approach big attacks. Specifically, using a threshold of 49, *alpha* = .924, 99% CI [.908, .940], beta = .878, 99% CI [.862, .894]; using a threshold of 31, *alpha* = .949, 99% CI [.933, .965], *beta* = .900, 99% CI [.885, .915]; using a threshold of 14, *alpha* = .958, 99% CI [.940, .976], *beta* = .954, 99% CI [.936, .972]; using a threshold of 9, *alpha* = 1.001, 99% CI [.982, 1.020], *beta* = 1.012, 99% CI [.992, 1.032].

## Discussion

In the series of incidents between big attacks (big attacks were defined as attacks with 21 or more victims killed), the lethality of a group’s attacks tended to first decrease following a big attack and then increase prior to a big attack. Specifically, beta distribution analyses showed that attacks that occur closer in time to the flanking big attacks tend to kill more people than attacks that occur near the midpoint between big attacks. In addition, we analyzed fatalities using MRCM, which focused only the sequence of attacks without considering the actual time points at which they occur during the progression. These analyses showed that incidents early and late in the sequence of attacks between big attacks tend to inflict the highest number of fatalities, relative to the middle of these sequences. These patterns appeared more pronounced with higher thresholds for big attacks (e.g., leading up to attacks that result in 49 or more fatalities). However, these patterns did not hold among groups that never committed a big attack (i.e., in the series of attacks that do not lead up to a big attack).

We also examined the patterns of attack-frequency between big attacks. The beta distribution analyses suggested that attacks tend to occur more frequently just after and just before big attacks (as reflected by a higher density at these time points) relative to the midpoint between big attacks. Moreover, the observed U-shape of the density estimates suggested an even higher density of attacks just prior to a big attack than just after a big attack. In contrast, the beta distribution analyses of groups that never commit a big attack looked quite different. They showed more of a steady trend towards de-escalation throughout their entire progression of attacks.

We analyzed frequency with MRCM as well, which again focused only the sequence of attacks without considering the actual time points at which they occur during the progression. These analyses suggested that with each consecutive attack, the number of days between attacks grows shorter in the middle of the sequence but then grows longer as groups approach the end of the sequence. Thus, though the beta distribution showed that the attacks in a progression are most frequent towards the end–close in time to an upcoming big attack–the final attacks in this sequence tend to have more days between them than attacks in the middle of the sequence. The pattern among groups who never committed a big attack looked different. These groups took longer overall between attacks. Further, toward the end of their lifespan they showed a more pronounced increase in the number of days between attacks than we observed in progressions leading up to big attacks.

As mentioned, the escalation patterns we have observed may have a number of explanations. It may be that killing triggers justification processes, desensitizes people, or produces material and/or psychological rewards that increase the likelihood of further killing [5]–[9]. Or perhaps with each attack, group members gain experience and thus increase the group’s ability to carry out big attacks.

In addition to escalation prior to big attacks, the data provide evidence for de-escalation after big attacks. As with escalation, there are various possible explanations for this pattern. For one, it may be that after big attacks, pressure, surveillance, or conflict from opponents (e.g., State opponents) may limit the resources terrorist groups have with which to operate. Second, these pressures may fragment groups, diminishing their ability to organize and carry out more deadly attacks [11]. Work in conjunction with those in various disciplines (e.g., political science, sociology, psychology, economics, physics) may help tease apart these explanations for escalation and de-escalation as well as contribute to the development of others.

Yet another path for future work is suggested by the evidence here that escalation before big attacks and de-escalation after big attacks occurred using varying definitions of big attacks. Because we varied the definition of big attacks, big attacks according to one threshold became small attacks using a higher threshold to define big attacks. For example, many big attacks defined with the threshold of 21 fatalities are included as small attacks in analyses defining big attacks with thresholds of 31 and 49. That similar patterns show up at many of these different thresholds for big attacks hints at a self-similar structure in these lead-up progressions [12]. Within large patterns of escalation and de-escalation we observed smaller patterns of de-escalation and escalation. Moreover, given that the data suggest smaller de-escalation and escalation cycles may be nested within larger cycles, it may be worth considering the possibility that escalation and de-escalation forces exert themselves simultaneously, though to different degrees.

## Conclusion

This work adds to a growing body of research modeling the timing of terrorist events [3]–[4], [11], [13]–[15]. Prior work suggests that big attacks occur in a relatively random fashion over the course of a group’s lifespan [3]–[4]. The present results, considered in conjunction with this previous work, suggest that though the occurrence of big attacks appears scattered somewhat randomly over the course of a group’s lifespan, the set of small-scale attacks that precede these big attacks has a more systematic pattern. It is important to note, however, that terrorist attack data are messy and the effects we find are modest in size. Thus, though the GTD is well-organized and relatively comprehensive, efforts should be made to examine these patterns using other sources of terrorism data, such as the RAND Database of Worldwide Terrorism Incidents (http://www.rand.org/nsrd/projects/terrorism-incidents.html).

Despite the modest size of the effects and the noise inherent in analyses of real-world data such as these, we believe these analyses have value given the importance of the topic and may provide leverage for deeper exploration. For example, future research may examine factors that moderate whether escalation precedes big attacks. Future research may also build on the present descriptive analyses with models suited for prediction or forecasting. In addition, future research might take a different approach to modeling the timing of attacks. For example, though we scaled lead-up progressions from 0 to 1, one might also approach these data by examining fatalities (or frequency of attacks) by week or by month. Finally, it may be worth applying the present escalation model to the examination of killing in other contexts such as in war, genocide, and gang violence.
